# Supplementary material for: Detection of diagnostic and prognostic methylation-based signatures in liquid biopsy specimens from patients with meningiomas
Source: Nat Commun. 2023 Sep 13;14:5669. doi: 10.1038/s41467-023-41434-z (PMC10499807; doi:10.1038/s41467-023-41434-z)
Supplement: Supplementary file 1 — Supplementary Information [file 41467_2023_41434_MOESM1_ESM.pdf]

Detection of Diagnostic and Prognostic Methylation-based Signatures in Liquid Biopsy Specimens from Patients with Meningiomas

Grayson A. Herrgott[1], James M. Snyder[1], Ruicong She[2], Tathiane M. Malta[1], Thais S. Sabedot[1], Ian Y. Lee[1], Jacob Pawloski[1], Guilherme G. Podolsky-Gondim[3], Karam P. Asmaro[1], Jiaqi Zhang[2], Cara E. Cannella[2], Kevin Nelson[1], Bartow Thomas[1], Ana C. deCarvalho[1], Laura A. Hasselbach[1], Kelly M.Tundo[1], Rehnuma Newaz[1], Andrea Transou[1], Natalia Morosini[1], Victor Francisco[1], Laila M. Poisson[1,2], Dhananjay Chitale[4], Abir Mukherjee[4], Maritza S. Mosella[1], Adam M. Robin[1], Tobias Walbert[1], Mark Rosenblum[1], Tom Mikkelsen[1], Steven Kalkanis[1], Daniela P. C. Tirapelli[3], Daniel J. Weisenberger[5], Carlos G. Carlotti Jr[3], Jack Rock[1], Ana Valeria Castro[1,6]*, Houtan Noushmehr[1,6]*

[1]Department of Neurosurgery, Omics Laboratory, Hermelin Brain Tumor Center, Henry Ford Health, Detroit, MI, USA

[2] Department of Public Health, Biostatistics, Henry Ford Health, Detroit, MI, USA

[3]Department of Neurosurgery, Ribeirao Preto Medical School, University of Sao Paulo, Ribeirao Preto, SP, Brazil

[4]Department of Pathology, Henry Ford Health, Detroit, MI, USA

[5]Department of Biochemistry and Molecular Medicine, Keck School of Medicine, University of Southern California, Los Angeles, CA 90033, USA.

[6] Department of Physiology, Michigan State University, E. Lansing, MI, USA

***Corresponding authors**
Ana Valeria Castro - acastro1@hfhs.org
Houtan Noushmehr - hnoushm1@hfhs.org

Supplementary Methods

Serum collection and processing

Peripheral blood (15 mL) was drawn from each subject at the time of surgical procedure prior to tumor excision. Serum samples were separated within 1 hour from collection by centrifugation at 1,300 x g for 10 minutes at 20°C; aliquoted into 2 mL cryovials and stored at -80°C until processing.

DNA isolation, quantification, quality control and DNA methylation data generation

DNA from serum samples (cfDNA) was extracted from 1.0-6.8 mL aliquots of serum and ~50 mg (min-max:10-80mg) of tissue using the Quick-cfDNA Serum & Plasma Kit according to the manufacturer's protocol (Zymo Research - catalog #D4076). DNA concentration was measured with Qubit (Thermo Fisher Scientific). The concentration of cfDNA in the serum was calculated by dividing the total amount of cfDNA extracted by the amount of serum used for extraction (ng/µL).

DNA Methylation using MethyLight (MS-qPCR) Technology

Assay Designs

The genomic DNA sequences 600 bp upstream and downstream (1,200 bp total) of the targeted CpG for nine Illumina EPIC probes were obtained from the Illumina EPIC probe manifest (www.illumina.com). The specific EPIC probes are: cg01181584, cg03845383, cg04208666, cg12942155, cg21245981, cg21699881, cg01465838, cg08402107 and cg25112312. Each DNA region was bisulfite converted in silico by replacing all non-CpG cytosine nucleotides with thymines. MethyLight primers and probes were designed to include the Illumina EPIC CpG dinucleotide assayed in each probe sequence. The primers and probes are listed in Supplementary Data 5. Each probe is 5' labeled with FAM and 3' labeled with either black hole quencher (BHQ-1) or minor groove binding non-fluorescent quencher (MGBNFQ) as indicated in the table. Primer designs were performed as described in Campan et al. [1]. BHQ-1 probes were synthesized by BioSearch Technologies (Petaluma, CA USA) and MGBNFQ probes were synthesized by ThermoFisher Scientific (Waltham, MA USA).

MethyLight assay data production

As a secondary method to detect methylated DNA in a small cohort of tissue, serum and plasma meningioma samples (n=12, n=11 and n=7, respectively) we employed the MethyLight assay. This is a highly sensitive and quantitative method that uses TaqMan-based real-time PCR assay using bisulfite-converted DNA as a template[1]. We used sequence-specific primers and probes for

nine DNA regions across each sample type. A control reaction targeting deaminated ALU repeats (HB-313) was used to correct for sample input amounts and a DNA sample treated with M.SssI methylase was used as a methylated reference sample. DNA methylation values for each sample and assay were calculated as Percent Methylated Reference (PMR) [1].

Whole Genome Bisulfite Sequencing (WGBS) of cfDNA samples

cfDNA samples isolated from serum were bisulfite converted using the Zymo EZ DNA methylation kit (Zymo Research, Irvine, CA USA) at the USC Molecular Genomics Core (Los Angeles, CA) as described by the manufacturer. Libraries were made and amplified using the Zymo-Seq cell free DNA WGBS library kit as recommended by the manufacturer. Amplified libraries were analyzed using Agilent BioAnalyzer 2100 technology and were cleaned using 0.8X beads to remove residual primer-dimers. Libraries were pooled to a 4uM concentration and sequenced on an Illumina MiSeq v2 micro 300bp flow cell to confirm accurate representation of each library in the pool. The pool concentration was adjusted to 2uM and then sequenced on an Illumina NextSeq2000 P3 200 bp flow cell.

DNA methylation preprocessing

DNA methylation array data was processed with the minfi package in R. Raw signal intensities were extracted from the *.idat files using the function 'readmetharray.exp' with appropriate hg38 annotations and corrected for background fluorescence intensities and red-green dye-bias using the function 'preprocessIllumina' [2]. Beta-values were calculated as (M/(M+U)), in which M and U refer to the (pre-processed) mean methylated and unmethylated probe signal intensities, respectively, using the function 'getBeta'. Measurements in which the fluorescent intensity was not statistically significant above background signal (detection p-value > 10^{-16}) were removed from the data set. Before the analysis, we removed probes designed for sequences with known polymorphisms (SNP), probes with poor mapping quality and the X and Y chromosomes (a complete list of masked probes is provided by Zhou et al.) [3].

Technical validation

WGBS paired with EPIC arrays were performed in 10 serum paired specimens from a new set of patients with meningiomas (predicted high risk: n=7 and low risk: n=3). Whole genome bisulfite sequencing data quality control was conducted following previously described standard procedures [4]. Briefly, quality of sequences was obtained through FastQC (version 0.11.9). Fragments at 3' and 5' ends and adapter sequences (phred score <20) were removed using

Trim_galore (version 0.6.6). Reads were then aligned to the human genome (hg38), duplicates were removed and DNA methylation was extracted through functions available in the Bismark pipeline (version 0.21.0), using default parameters [5].

Comparison of CpG site DNA methylation between EPIC-array and WGBS

CpG sites shared between WGBS and EPIC array assays were defined using genomic coordinates. To filter WGBS sites for downstream analysis, first, we calculated the average coverage, i.e., the sum of methylated and unmethylated cytosines for individual genomic positions, observed across all samples ($n=10$, $\mu=2.5$). Then, taking this value into account, we selected CpG sites with a coverage $\geq$ 3X depth to ensure data quality across comparisons [6,7]. Pearson correlations and absolute value differences were calculated between WGBS CpG-level methylation percentage values (% DNA methylation/100) and EPIC array beta-values [8], across common CpG sites defined according to genomic coordinates. When considering the absolute value differences, we separated them by value distribution quantiles, highlighting those CpGs which showed the highest value similarity between WGBS and EPIC technologies (Q1: Δ DNA methylation <0.11).

Circulating cell-free DNA profiling through WGBS, across 10 samples composed of MNG predicted to have high or low risks to recur, displayed on average 22,647,947 CpGs and coverage of 2.56X depth, prior to any coverage-based filtering. Correlations across unfiltered CpG sets between WGBS and unmasked paired EPIC arrays ranged between $\rho=0.816$ and 0.863 ($p\leq 2.2e-16$) (Supplementary Table S2). Following all quality control, WGBS captured an average of 9,256,306 CpGs with total coverage $\geq$3X depth across the 10 cfDNA samples. An average of 239,333 CpG sites were shared between EPIC array and filtered WGBS, with genome-wide DNA methylation correlations ranging between $\rho=0.841$ and 0.896 ($p\leq 2.2e-16$) (Supplementary Table S2).

Unsupervised Analysis

We retrieved 573k non-zero and unmasked CpG probes shared across meningioma (MNG) and non-meningioma (non-MNG) serum methylomes. Next, we generated a three-dimensional (3D) genome-wide Principal Component Analysis (PCA) of the mean methylome levels across all serum samples from patients with distinct tumor types and non-neoplastic brain diseases, using the function ‘prcomp’ (package: stats v3.6.0) (Figure 1a). We also retrieved the 1,000 most variably methylated probes across the meningioma serum sample cohort (n=63), which were used in the downstream agglomerative hierarchical clustering and visualized in a heatmap (Figure

2a).

Molecular Deconvolution

In order to estimate the immune cell proportions across meningioma risk-related subgroups and DNA methylation-based k-clusters, we used the MethylCIBERSORT approach [9,10]. In short, a reference cell matrix was compiled using publicly available cell type profiles [9,10] with the resulting signature set formulated through the limma-based wrapper function to fit a series of linear models for pairwise comparisons, as provided by and further detailed in Chakravarthy et al. [9,10]. Once relevant signatures for the candidate cell types were isolated, the standalone program MethylCIBERSORT [9,10] was used to estimate relative cell contributions to each liquid biopsy or tissue sample. Cell proportions per sample type were multiplied by a factor of 100 to convert to full number ranges.

Comparison of random forest classifier with other machine learning methods.

We compared the performance of our Random Forest (RF)-based classifier (i.e., diagnostic-Meningioma Epigenetic Liquid Biopsy [d-MeLB] model) to alternative machine learning and regression approaches to classify specimens as MNG or non-MNG - First, to isolate the particular advantages of RF, machine learning linear discriminant analyses (LDA) were conducted across 1,000 iterations applying the identical randomized cohort, feature definition and model selection procedures. Second, we formulated an unsupervised RF methodology and used the similar methylated probes (SMPs) resulting from similarity measures (dimension reduction) between meningioma tissue and serum, to investigate the efficiency of employed supervised methods compared to less specialized methods. Third, we constructed an Extreme Gradient Boosting algorithm (package: XGBoost v1.7.4) utilizing signatures derived within d-MeLB (n=25 CpGs) [11]. Generated algorithms' hyper-parameters were optimized using fivefold cross-validation resampling techniques (function 'xgb.cv'), allowing for experimentation and isolation of optimal parameters for diagnostic purposes. Finally, we formulated univariate and multivariate linear regressions using naive baseline mean-methylation statistics (whole-genome: n=~573k CpGs and/or meningioma-specific: n=98 DMPs) to train classifiers. The performance of these alternative classifiers was trained and validated across identical cohorts to the d-MeLB classifier.

Identification of biologically relevant and equivalent DNA methylation markers between tissue and serum

We applied the prognostic-Meningioma Epigenetic Liquid Biopsy (p-MeLB) model to classify the Choudhury methylome cohort (n=185) into high and low risk groups and intersected these results with equivalent prognostic groups detailed throughout their publication, i.e. hypermitotic and merlin-intact groups [12]. We performed supervised analyses across the transcriptome of the resulting groups (high-risk hypermitotic MNG and low-risk merlin intact MNG) to identify differentially expressed genes (DEG) using the DESeq2 (v1.36.0) package [13]. We mapped the resulting DEGs to CpGs located in their regulatory elements (promoters and enhancers) and selected those CpGs which simultaneously presented differential prognostic-group DNA methylation levels and negative correlation with the expression of putative target genes, to infer a potential epigenetic regulation. Next, we explored these DMPs across the serum methylome to identify CpGs which possessed similar tissue-DNA methylation levels and simultaneously distinguished serum specimens by p-MeLB recurrence risk.

In a complementary manner, serum DMPs derived from comparisons of high and low risk groups were mapped to tissue derived DEGs (n=32 PGPs). These DMPs were analyzed for concordant DNA methylation across tissue groups (e.g., serum groups: hypermethylation in high-risk vs low risk; tissue groups: hypermethylation in high-risk hypermitotic vs low-risk merlin), and inverse correlation with gene behavior (e.g., hypermethylation and downregulation of gene). Then, we conducted gene set enrichment analyses (GSEA) through the Ingenuity Pathway Analysis (IPA) platform, identifying relevant diseases and biological processes related to these concordantly methylated recurrence risk-related DMP-genes.

Clinicopathological and molecular features across MNG subgroups

Categorical variables (n=number of samples) were converted to proportions (e.g., $n_{category1}/n_{category2}$), with odds ratios and 95% confidence intervals calculated between the proportions of high to low-risk groups and assigned a statistical significance ($p \leq 0.05$). For discrete features, we calculated between-group differences across the aforementioned groups (mean difference), associated 95% confidence intervals, and assigned statistical significances ($p \leq 0.05$). As reference, we calculated the distributions of each feature across the whole meningioma cohort (serum or tissue).

Additionally, we generated a z-score heatmap to delineate the enrichment or depletion of specific categorical and continuous variables related to clinicopathological, imaging and molecular features across DNA methylation groups (k-means clusters).

References

1. Campan, M., Weisenberger, D. J., Trinh, B. & Laird, P. W. Methylight and digital methylight. *Methods Mol. Biol.* **1708**, 497–513 (2018).
2. Triche, T. J., Weisenberger, D. J., Van Den Berg, D., Laird, P. W. & Siegmund, K. D. Low-level processing of Illumina Infinium DNA Methylation BeadArrays. *Nucleic Acids Res.* **41**, e90 (2013).
3. Zhou, W., Laird, P. W. & Shen, H. Comprehensive characterization, annotation and innovative use of Infinium DNA methylation BeadChip probes. *Nucleic Acids Res.* **45**, e22 (2017).
4. Wreczycka, K. *et al.* Strategies for analyzing bisulfite sequencing data. *J. Biotechnol.* **261**, 105–115 (2017).
5. Krueger, F. & Andrews, S. R. Bismark: a flexible aligner and methylation caller for Bisulfite-Seq applications. *Bioinformatics* **27**, 1571–1572 (2011).
6. Chan, K. C. A. *et al.* Noninvasive detection of cancer-associated genome-wide hypomethylation and copy number aberrations by plasma DNA bisulfite sequencing. *Proc Natl Acad Sci USA* **110**, 18761–18768 (2013).
7. Li, W. *et al.* CancerDetector: ultrasensitive and non-invasive cancer detection at the resolution of individual reads using cell-free DNA methylation sequencing data. *Nucleic Acids Res.* **46**, e89 (2018).
8. Shu, C., Zhang, X., Aouizerat, B. E. & Xu, K. Comparison of methylation capture sequencing and Infinium MethylationEPIC array in peripheral blood mononuclear cells. *Epigenetics Chromatin* **13**, 51 (2020).
9. Moss, J. *et al.* Comprehensive human cell-type methylation atlas reveals origins of circulating cell-free DNA in health and disease. *Nat. Commun.* **9**, 5068 (2018).
10. Chakravarthy, A. *et al.* Pan-cancer deconvolution of tumour composition using DNA methylation. *Nat. Commun.* **9**, 3220 (2018).
11. Chen, T. & Guestrin, C. XGBoost: A Scalable Tree Boosting System. in *Proceedings of the 22nd ACM SIGKDD International Conference on Knowledge Discovery and Data Mining - KDD '16* 785–794 (ACM Press, 2016). doi:10.1145/2939672.2939785.
12. Choudhury, A. *et al.* Meningioma DNA methylation groups identify biological drivers and therapeutic vulnerabilities. *Nat. Genet.* **54**, 649–659 (2022).
13. Love, M. I., Huber, W. & Anders, S. Moderated estimation of fold change and dispersion for RNA-seq data with DESeq2. *Genome Biol.* **15**, 550 (2014).
14. Bayley, J. C. *et al.* Multiple approaches converge on three biological subtypes of meningioma and extract new insights from published studies. *Sci. Adv.* **8**, eabm6247 (2022).

15. Gao, F. *et al.* DNA methylation in the malignant transformation of meningiomas. *PLoS ONE* **8**, e54114 (2013).

Supplementary Table S1. Matching liquid biopsy serum and plasma correlation coefficients across continuous measures (genome-wide methylation and estimated immune proportions) and classifier concordances.

			Continuous Correlation (ρ)		Classifier Concordance	
Pair #	Sample IDs	Cohort	GW Methylation	Immune Proportions	d-MeLB	p-MeLB
1	HBTC-ADD-03/04	Additional MNG	0.95**	0.7**	No	Yes
2	HBTC-ADD-05/06		0.93**	0.81**	Yes	Yes
3	HBTC-ADD-07/08		0.93**	0.98**	Yes	Yes
4	HBTC-ADD-09/10		0.89**	0.93**	Yes	No
5	HBTC-ADD-11/12		0.92**	1.00**	Yes	Yes
6	HBTC-ADD-19/20		0.96**	1.00**	Yes	No
7	HBTC-ADD-21/22		0.91**	1.00**	No	Yes
8	HBTC-ADD-23/24		0.95**	0.95**	Yes	Yes
9	HBTC-ADD-25/26		0.94**	0.87**	Yes	Yes
10	HBTC-ADD-27/28		0.91**	0.97**	Yes	No

Note: **: two-sided t-test p-value ≤ 2.2E-16; GW: Genome-Wide; MNG: Meningioma; d-MeLB: Diagnostic-Meningioma epigenetic Liquid Biopsy; p-MeLB: Prognostic-Meningioma epigenetic Liquid Biopsy.

Supplementary Table S2. Pearson correlative analysis between the entire and filtered genome across WGBS and EPIC array.

Sample ID	WGBS Captured CpGs	Entire Genome			WGBS Filtered Total CpGs	Filtered Genome (Coverage > 3x Depth)		
		Shared CpGs	Pearson's Rho	95% CI		Shared CpGs	Pearson's Rho	95% CI
HBTC-ADD-14	21,998,645	560,985	0.835**	(0.834, 0.836)	8,307,798	203,551	0.875**	(0.874, 0.876)
HBTC-ADD-15	22,535,927	576,307	0.857**	(0.856, 0.858)	8,665,122	219,708	0.886**	(0.885, 0.887)
HBTC-ADD-16	23,733,248	611,099	0.815**	(0.814, 0.816)	10,952,759	273,366	0.852**	(0.851, 0.853)
HBTC-ADD-17	21,443,877	542,031	0.851**	(0.85, 0.852)	7,027,928	170,687	0.889**	(0.888, 0.89)
HBTC-ADD-18	23,327,911	607,929	0.863**	(0.862, 0.864)	10,071,012	266,299	0.896**	(0.895, 0.896)
HBTC-ADD-19	22,805,199	596,564	0.837**	(0.837, 0.838)	8,736,738	233,598	0.881**	(0.88, 0.882)
HBTC-ADD-21	23,733,248	638,818	0.857**	(0.855, 0.856)	12,405,916	343,859	0.883**	(0.882, 0.883)
HBTC-ADD-23	23,583,202	610,099	0.836**	(0.835, 0.837)	11,294,592	296,362	0.859**	(0.858, 0.86)
HBTC-ADD-26	23,209,791	590,351	0.816**	(0.816, 0.817)	10,024,292	249,839	0.841**	(0.841, 0.842)
HBTC-ADD-27	20,108,426	530,278	0.822**	(0.821, 0.823)	5,076,899	136,061	0.871**	(0.87, 0.872)

Note: WGBS: whole genome bisulfite sequencing; CI: Confidence Interval; **: two-sided t-test p-values $\leq$ 2.2e-16.

Supplementary Table S3. Performance measurements of d-MeLB compared with other machine learning- or regression-based classifier approaches, formulated in-house.

	Machine Learning				Logistic Regression: Univariate & Multivariate		
	d-MeLB [RF]	DR [RF]	LDA	XGBoost	GW	Supervised	GW & Supervised
Signature Set (n):	25 CpGs	8,869 SMPs	21 CpGs	25 CpGs	~573k CpGs	98 DMPs	~735k CpGs & 98 DMPs
Serum-based Model Selection Set (n=23)							
AUC:	1.00	0.913	0.87	0.99	N/A	N/A	N/A
Total Independent Validation (Serum & Plasma; n=122)							
ACC (%):	81.1	72.1*	63.1*	81.9	57.4*	73.0	74.6
SE (%):	73.8	33.3*	38.1*	73.8	76.2	88.1	90.5
SP (%):	85.0	92.5	76.3*	86.3	47.5*	65.0*	66.3*
CUI (+):	0.532	0.233	0.174	0.545	0.329	0.501	0.529
CUI (-):	0.732	0.671	0.535	0.744	0.376	0.593	0.616
Immune-Based Validation (Serum & Whole Blood; n=59):							
ACC (%):	93.2	100	27.1	89.8	88.1	100	100

Note: All statistical comparisons (two-sided Fisher's Exact test) are conducted between d-MeLB and the indicated classifier. RF: Random Forest; DR: Dimension Reduction; CUI (+): Clinical Utility Index for positive cases; CUI (-): Clinical Utility Index for negative cases; LDA: Linear Discriminant Analysis; DMPs: differentially methylated probes; *: comparison to d-MeLB performance is statistically significant at two-sided Fisher's Exact Test p-value $\leq .05$.

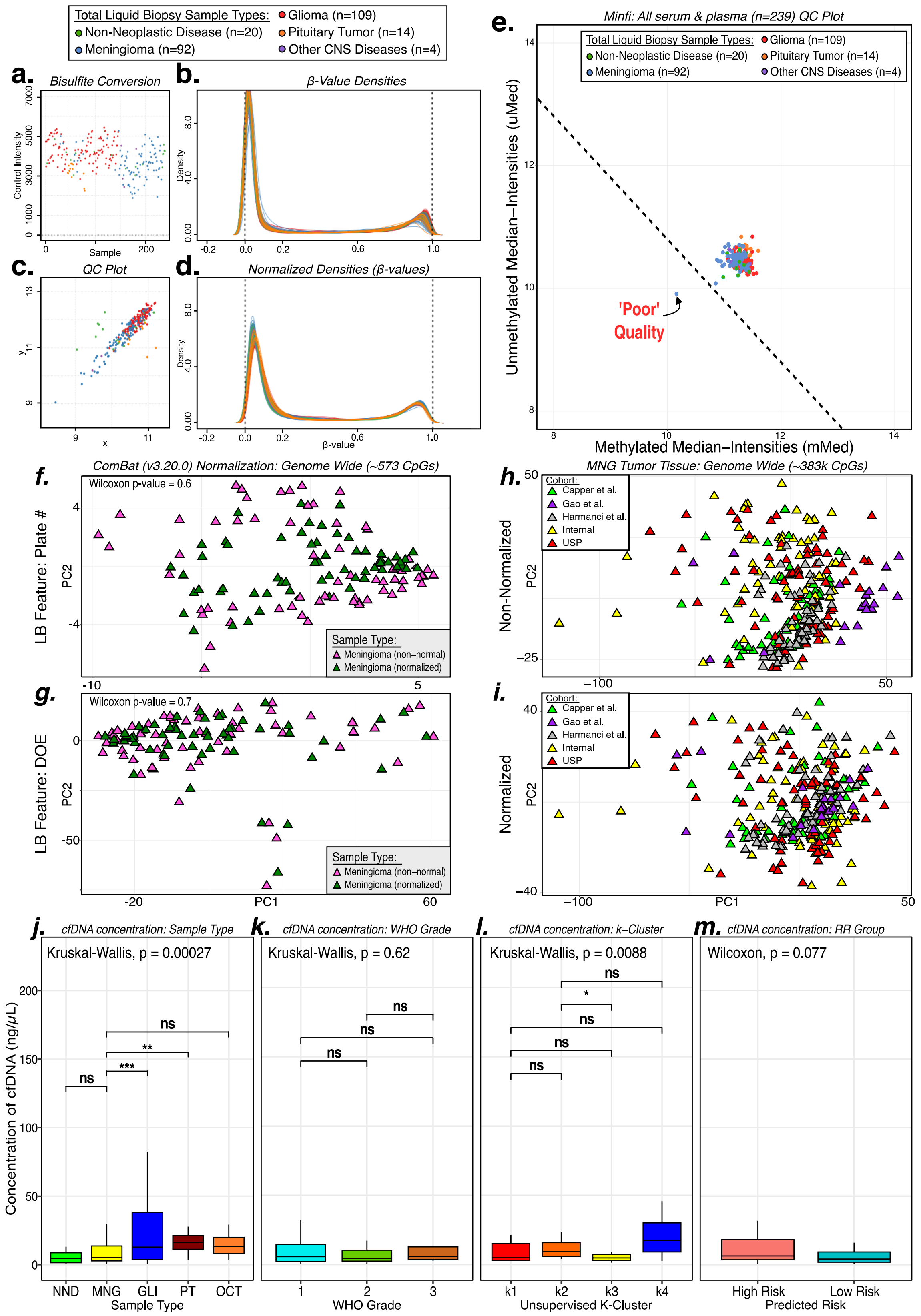

Supplementary Figure S1. Liquid biopsy cell free (cf)-DNA quality features in the meningioma (MNG, n=93) and non-MNG serum cohorts (n=141). a-d Preprocessed methylation array data visualization (EPIC) using shinyMethyl v3.16. Samples are color coded by sample type. Note: CNS: Central Nervous System. **e** Evaluation of median methylation level intensity (log) in both methylated & unmethylated channels using Minfi package. Notes: QC: quality control. **f-g** Genome-wide principal component analysis of non-normalized and normalized MNG samples according to plate (**f**) and cfDNA extraction date (DOE) (**g**). **h-i** Genome wide principal component analysis of non-normalized (**h**) and normalized (**i**) MNG tumor tissue according to institution. **j-m** Comparison between serum cfDNA concentration (ng/mL) across (**j**) sample types [exact p-values: Meningioma vs Glioma: p=0.00099; Meningioma vs Pituitary tumors: p=0.005], (**k**) MNG WHO grades, (**l**) k-means methylation clusters [exact p-values: k2 vs k3: 0.012] and (**m**) predicted recurrence risk groups (Kruskal-Wallis; Wilcoxon test; ns: non-significant; *: p<0.05, **: p<0.01, ***: p<0.001; ****: p<0.0001). Box plots - data are presented as median and upper (75%) and lower (25%) quartiles. Whiskers represent minimum to maximum values, excluding outliers. Note: NND: Non-neoplastic disease; MNG: Meningioma; GLI: Glioma; PT: Pituitary Tumors; OCT: Other CNS Tumors.

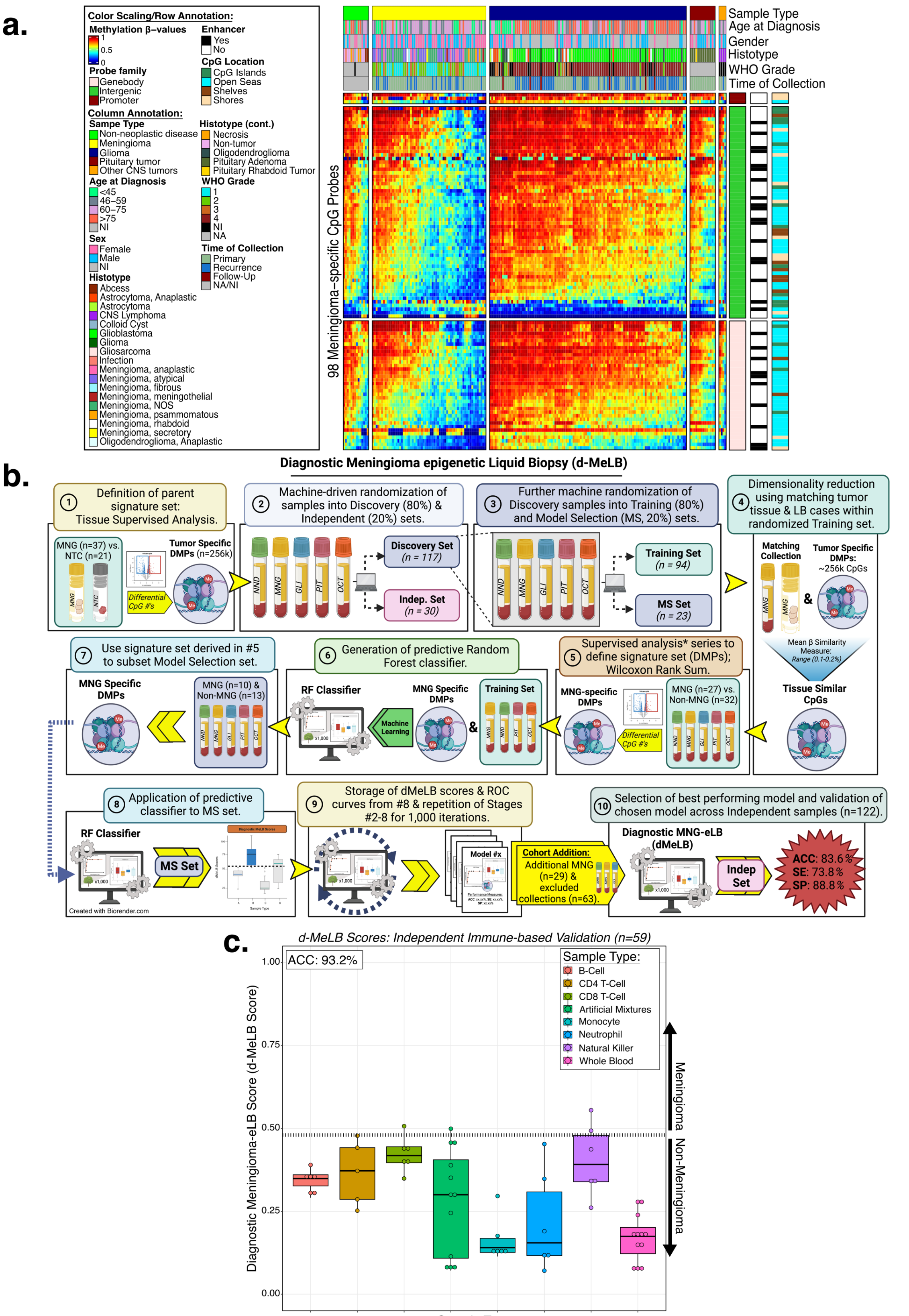

Supplementary Figure S2. Meningioma-specific DNA methylation markers and diagnostic-oriented analyses and machine learning methodologies. **a** Heatmap displaying the methylation β-values of MNG-specific differentially methylated probes (DMPs) in relation to healthy meninges and other CNS entity samples (n=98) across serum samples, associated clinicopathological features and genomic annotations (Column: clinicopathological features; Row: genomic location/probe family annotation). Note: NA: non-applicable; NI: Non-informed. **b** Schematic representing the stepwise development of the diagnostic-Meningioma Epigenetic Liquid Biopsy (d-MeLB) machine learning classifier. Note: MNG: meningioma; '*': untreated MNG population, non-MNG are sampled from the total non-MNG cohort at a rate of 0.8, with exclusions for glioblastoma. Note: NTC: non-tumor control; MS: model selection; RF: Random Forest; DMP: differentially methylated probes; ROC: receiver operating characteristic. **c** Distribution of the d-MeLB scores across an independent immune-focused cohort (n=59) (Dashed line: MeLB cutoff score). Box plots - data are presented as median and upper (75%) and lower (25%) quartiles. Whiskers represent minimum to maximum values, excluding outliers. Upper left corner: performance measures. Note: ACC: Accuracy.

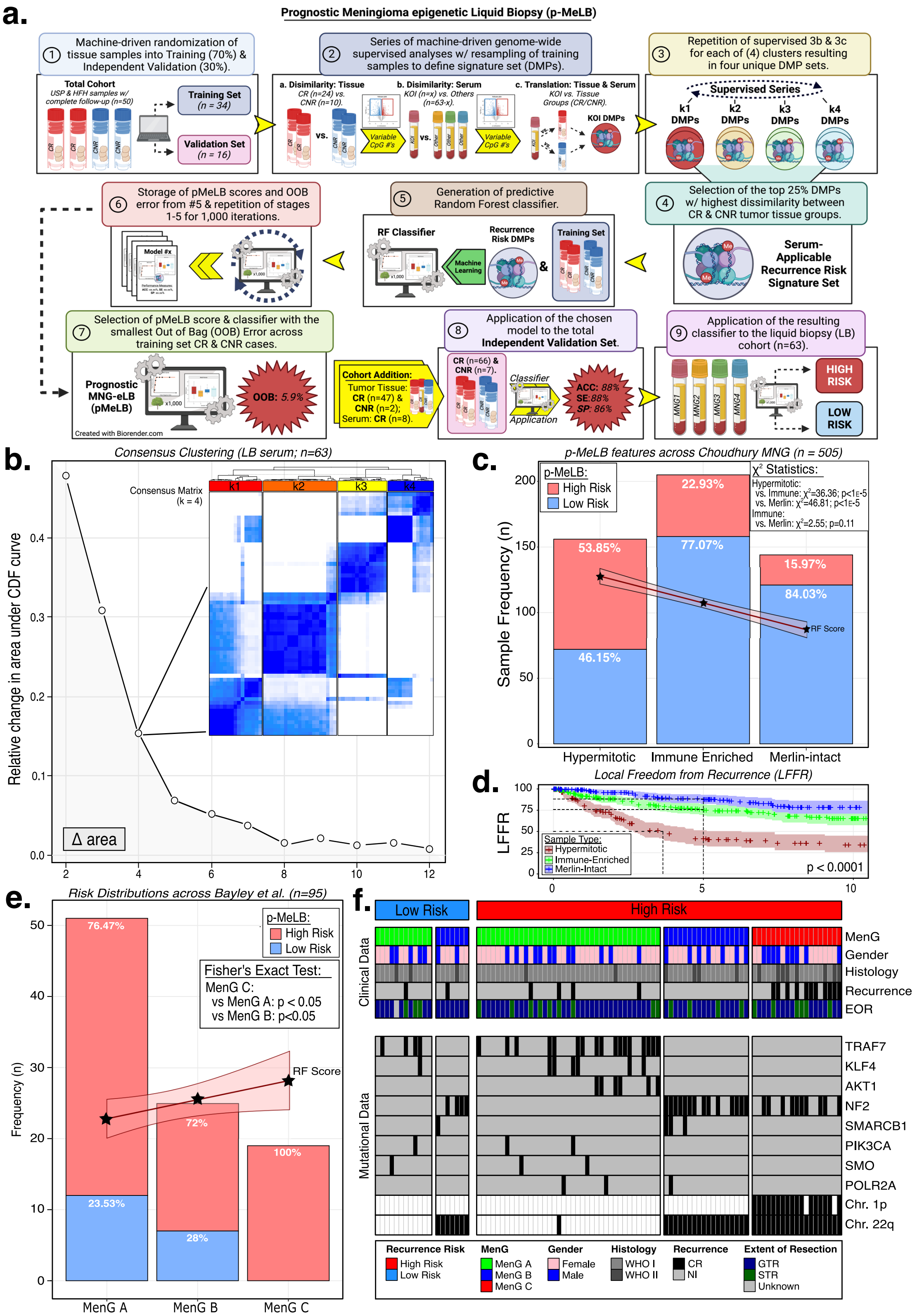

Supplementary Figure S3. Prognostic-Meningioma Epigenetic Liquid Biopsy (p-MeLB) development and comparison with other DNA methylation-based tissue-derived prognostic classifiers. **a** Schematic representing the stepwise development of the p-MeLB machine learning prediction model. Note: USP: University of Sao Paulo; HFH: Henry Ford Health; CNR: Confirmed No Recurrence, CR: Confirmed Recurrence; DMP: differentially methylated probes; KOI: k-means cluster of interest; RF: Random Forest; OOB: Out-of-Bag; ACC: accuracy; SE: sensitivity; SP: specificity. **b** Consensus matrix heatmap and delta area curves used to determine the optimal number of k-means hierarchical clusters (ConsensusClusterPlus) that present enrichment for prognostic features. Note: CDF: cumulative distribution function. **c** Proportion distribution (stacked barplot) of p-MeLB-derived recurrence risk predictions (high and low risk) across Choudhury prognostic tissue groups (hypermitotic, immune-enriched and merlin-intact, n=505). Relationship of groups and mean p-MeLB score is depicted in a standard regression line, with 95% confidence interval upper and lower limits. Test statistics: two-sided chi-squared test of independence. **d** Kaplan-Meier curves displaying overall local freedom from recurrence (LFFR) attributed to the Choudhury [12] prognostic group. Survival curves are depicted with 95% confidence intervals (lower and upper limits) for point estimates; comparisons of median survival time in both recurrence risk groups were conducted using log-rank tests (p<0.0001). **e** Proportion distribution of p-MeLB-derived recurrence risk predictions (high and low risk) across Bayley methylation-based prognostic groups [14] (MenG A, B, C; n=95). Relationship of groups and mean p-MeLB score is depicted in a standard regression line, with 95% confidence intervals (upper and lower limits; two-sided Fisher's Exact test). Note: MenG: Bayley Meningioma Group. **f** Oncoplot displaying the distribution of methylation groups, clinicopathological and mutational/cytogenetic data across the Bayley cohort [14] classified by p-MeLB. Note: EOR: Extent of resection; GTR: gross total resection; STR: subtotal resection; CR: Confirmed Recurrence; NI: non-informed.

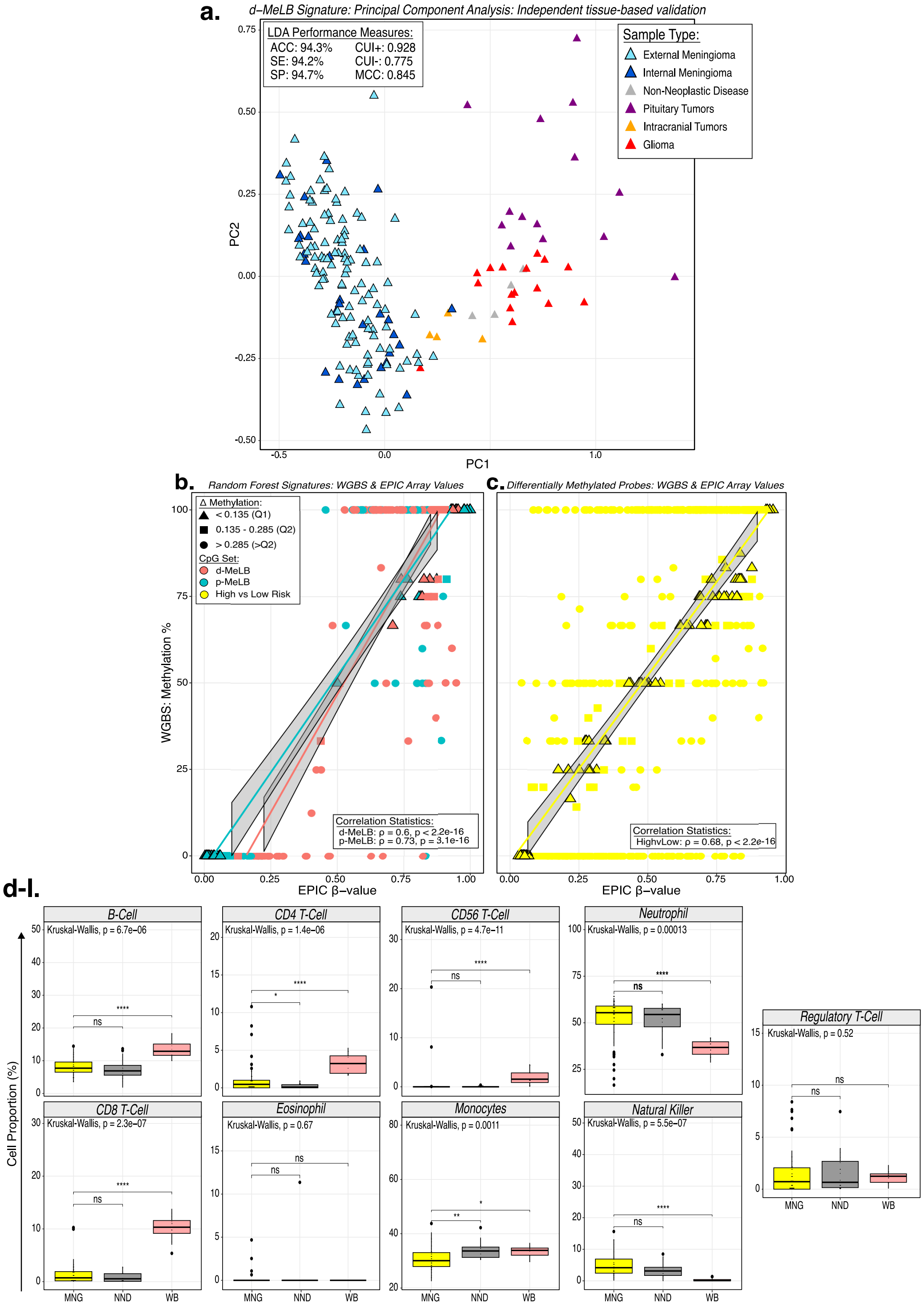

Supplementary Figure S4. Linear Discriminant Analysis and technical validations across tissue samples and DNA profiling methods, respectively, using d- and p-MeLB signatures and meningioma-specific immune cell types of estimations through a methylation-based deconvolution across cfDNA serum samples. **a** Principal component analysis (PCA) displaying the performance of the linear discriminant analysis (LDA) model to classify an independent tissue-based cohort of internal and external collections (n=176). LDA was trained using d-MeLB signatures (n=25 CpGs) across the visualized cohort. Upper left corner: performance measures. Note: LDA: linear discriminant analysis; SE: sensitivity; SP: specificity; CUI: Clinical Utility Index; MCC: Matthew's Correlation Coefficient; **b-c** Scatterplots depicting the observed relationship between WGBS methylation percentage (%) and EPIC array β-values across d- and p-MeLB model Random Forest signatures (**b**) and risk-related DMP sets (high and low risk) (**c**). CpG signatures are colored by their respective group and shaped by their absolute change in methylation (Δ methylation) between profiling techniques. Linear relationships are depicted with 95% confidence intervals (lower and upper limits). Bottom right: Pearson's correlation coefficients. **d-l** Comparisons between the estimated immune cell types-specific proportions (%) across sample types (Kruskal-Wallis; Wilcoxon rank sum test; ns: non-significant; *: p<0.05, **: p<0.01, ***: p<0.001; ****: p<0.0001). Note: MNG: Meningioma; NND: Non-neoplastic Diseases; WB: Whole Blood. Box plots - data are presented as median and upper (75%) and lower (25%) quartiles. Whiskers represent minimum to maximum values, excluding outliers. Upper left corner: performance measures. Exact p-values: B-Cell - Meningioma vs Whole Blood: p=2.2e-06; CD4 T-Cell - Meningioma vs Non-Neoplastic Disease: p=0.048; Meningioma vs Whole Blood: p=8.3e-06; CD56 T-Cell - Meningioma vs Whole Blood: p=1.7e-10; CD8 T-Cell - Meningioma vs Whole Blood: p=1.2e-07; Monocytes - Meningioma vs Non-Neoplastic Disease: p=0.0014; Meningioma vs Whole Blood: p=0.016; Neutrophil - Meningioma vs Whole Blood: p=8.2e-05; Natural Killer - Meningioma vs Whole Blood: p=1.9e-07.

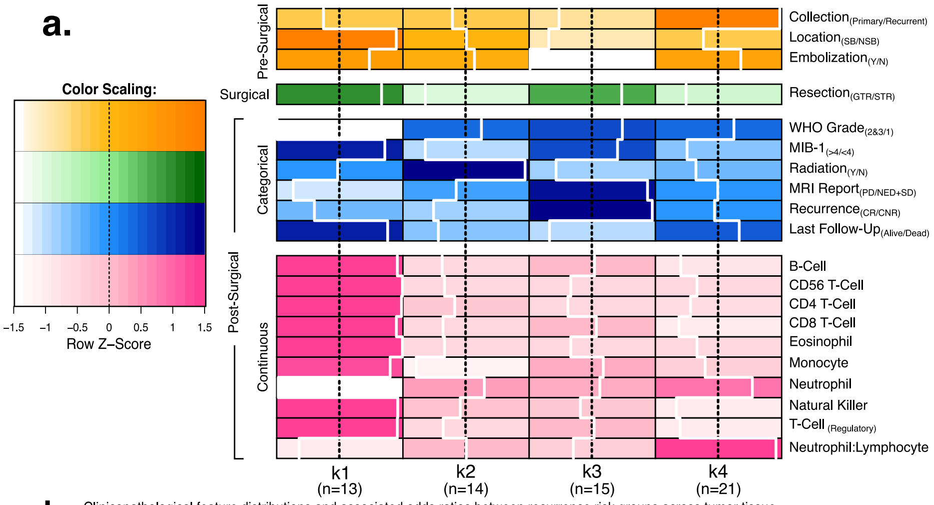

b. Clinicopathological feature distributions and associated odds ratios between recurrence risk groups across tumor tissue.

Characteristic	Reference (Whole Cohort) Ratio	High Risk Ratio	Low Risk Ratio	p
Collection (Initial/Recurrent)	**1.478**	**0.7**	**4.5**	**p≤0.005**
Location (SB/NSB)	0.3	0.185	0.539	*NS*
Resection (GTR/STR)	**2.294**	**1.333**	**9.5**	**p≤0.01**
WHO Grade (2&3/1)	6.125	7.75	4.5	*NS*
MRI Report (PD/NED+SD)	**0.857**	**2.1**	**0.167**	**p≤2E-04**
Recurrence (CR/CNR)	**0.857**	**16.5**	**0.467**	**p≤7E-07**

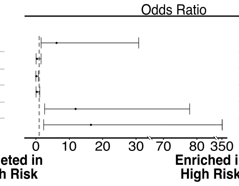

c. Immune proportion distributions and associated mean differences between recurrence risk groups across tumor tissue.

Characteristic	Reference (Whole Cohort) Mean (sd)	High Risk Mean (sd)	Low Risk Mean (sd)	p
B-Cell	0.134 (0.068)	0.138 (0.069)	0.127 (0.066)	*NS*
CD56 T-Cell	0.001 (0.009)	0.002 (0.011)	2.24E-04 (0.001)	*NS*
CD4 T-Cell	0.018 (0.033)	0.024 (0.039)	0.01 (0.016)	*NS*
CD8 T-Cell	0.012 (0.03)	0.0125 (0.023)	0.012 (0.039)	*NS*
Eosinophil	0.043 (0.024)	0.044 (0.024)	0.04 (0.023)	*NS*
Monocyte	**0.419 (0.067)**	**0.4 (0.057)**	**0.45 (0.073)**	**0.007**
Neutrophil	0.039 (0.039)	0.045 (0.037)	0.03 (0.042)	0.07
Natural Killer	0.144 (0.044)	0.141 (0.042)	0.132 (0.045)	0.1
T-Cell (Regulatory)	0.163 (0.051)	0.156 (0.045)	0.174 (0.058)	*NS*
NLR/100	0.183 (0.181)	0.213 (0.179)	0.135 (0.177)	0.08

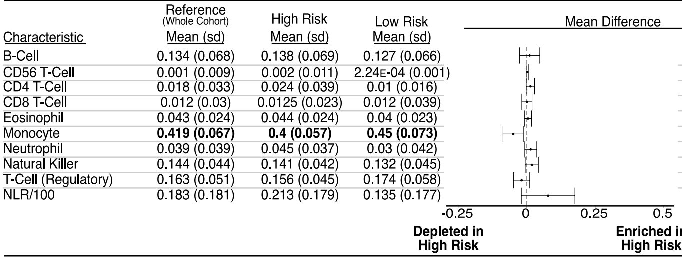

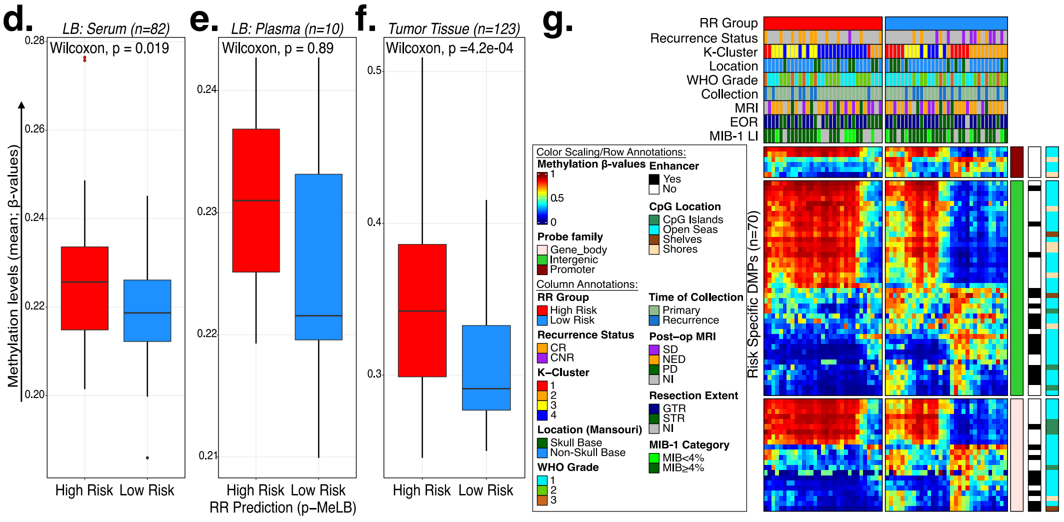

Supplementary Figure S5. Clinicopathological and molecular characterization of recurrence risk serum groups. **a** Proportion distribution of relevant clinicopathological, imaging and molecular features across k-clusters. Results are presented in the form of z-scores representing standard deviations (vertical white lines/row) from the row average (dashed lines). Feature enrichment/depletion highlighted in color gradient. Note: MRI: Magnetic Resonance Image. **b** Clinicopathological feature proportions and associated odds ratios derived from the comparison between meningioma serum samples predicted to present high or low recurrence risks (p-values: two-sided Fisher's Exact test; error bars: 95% confidence interval estimates). Reference column depicts the mean proportion of each feature across the whole cohort. Note: SB: Skull-base; NSB: Non-Skull Base; GTR: Gross Total Resection; STR: Subtotal Resection; PD: Progressive Disease; SD: Stable Disease; NED: Non-Enhancing Disease; CR: Confirmed Recurrence; CNR: Confirmed No Recurrence; Bolded features are those with observed statistical significance. **c** Immune cell proportions and associated mean differences derived from the comparison between meningioma tissue samples predicted to present high or low recurrence risks (error bars: mean difference 95% confidence interval; p-values: two-sided t-test). Reference column depicts the mean proportion across the whole cohort. Note: NLR: Neutrophil-Lymphocyte Ratio. Bolded features are those with observed statistical significance. **d-f** Comparison of the mean DNA methylation of CpG island probe sets reportedly associated with malignant transformation (Gao et al.) [15] across (**d**) serum, (**e**) plasma and (**f**) tissue specimens predicted to have high or low recurrence risk (two sided Wilcoxon rank-sum test). Box plots - data are presented as median and upper (75%) and lower (25%) quartiles. Whiskers represent minimum to maximum values, excluding outliers. Upper left corner: performance measures. Note: LB: liquid biopsy; RR: recurrence risk. **g** Heatmap displaying the β-values of risk-specific DMPs across serum samples (Column: clinicopathological features; Row: genomic location/probe family annotation). Note: RR group: recurrence risk prediction; MRI: magnetic resonance image; EOR: extent of resection, LI: labeling index; DMP: differentially methylated probes; SB: Skull-base; NSB: Non-Skull Base; GTR: Gross Total Resection; STR: Subtotal Resection; PD: Progressive Disease; SD: Stable Disease; NED: Non-Enhancing Disease; CR: Confirmed Recurrence; CNR: Confirmed No Recurrence.
