# Supplementary material for: Detection of diagnostic and prognostic methylation-based signatures in liquid biopsy specimens from patients with meningiomas
Source: Nat Commun. 2023 Sep 13;14:5669. doi: 10.1038/s41467-023-41434-z (PMC10499807; doi:10.1038/s41467-023-41434-z)
Supplement: Supplementary file 3 — Reporting Summary [file 41467_2023_41434_MOESM3_ESM.pdf]

nature portfolio

Corresponding author(s): Castro, Ana Valeria MD, PhD

Last updated by author(s): Aug 23, 2023

Reporting Summary

Nature Portfolio wishes to improve the reproducibility of the work that we publish. This form provides structure for consistency and transparency in reporting. For further information on Nature Portfolio policies, see our Editorial Policies and the Editorial Policy Checklist.

Statistics

For all statistical analyses, confirm that the following items are present in the figure legend, table legend, main text, or Methods section.

n/a	Confirmed	
[ ]	[x]	The exact sample size (n) for each experimental group/condition, given as a discrete number and unit of measurement
[x]	[ ]	A statement on whether measurements were taken from distinct samples or whether the same sample was measured repeatedly
[ ]	[x]	The statistical test(s) used AND whether they are one- or two-sided<br>*Only common tests should be described solely by name; describe more complex techniques in the Methods section.*
[ ]	[x]	A description of all covariates tested
[ ]	[x]	A description of any assumptions or corrections, such as tests of normality and adjustment for multiple comparisons
[ ]	[x]	A full description of the statistical parameters including central tendency (e.g. means) or other basic estimates (e.g. regression coefficient) AND variation (e.g. standard deviation) or associated estimates of uncertainty (e.g. confidence intervals)
[ ]	[x]	For null hypothesis testing, the test statistic (e.g. F, t, r) with confidence intervals, effect sizes, degrees of freedom and P value noted<br>*Give P values as exact values whenever suitable.*
[x]	[ ]	For Bayesian analysis, information on the choice of priors and Markov chain Monte Carlo settings
[x]	[ ]	For hierarchical and complex designs, identification of the appropriate level for tests and full reporting of outcomes
[ ]	[x]	Estimates of effect sizes (e.g. Cohen's d, Pearson's r), indicating how they were calculated

Our web collection on statistics for biologists contains articles on many of the points above.

Software and code

Policy information about availability of computer code

Data collection	R (v 3.6.1) package "GEOquery" (v2.68.0) for acquisition of publicly available datasets.
Data analysis	R (v 3.6.1) and mentioned packages (caret v6.0.94, ComBat v3.20.0, ComplexHeatmap v2.16.0, DESeq2 v1.36.0, MethylCIBERSORT v0.2, minfi v1.46.0, shinyMethyl v3.16, stats v3.6.0, Trim_galore v0.6.6, XGBoost v1.7.4). Other, unmentioned background packages include (AnnotationHub v3.8.0, annotatr v1.26.0, dplyr 1.1.2, GenomicRanges v1.52.0, ggpubr v0.6.0, ggplot2 v3.4.2, plot3D v1.4, parallel v4.3.0, rtracklayer v1.60.0, Rtsne v0.16, survival v3.5.5, tidyverse v2.0.0).<br>We have included custom syntax necessary for generation and application of our random forest algorithm to internal and publicly available liquid biopsy samples in a GitHub repository [https://github.com/gherrgo/eLB-Random-Forests.git], with raw methylation files located in a Mendeley Data repository as detailed in the Main Text (DOI: 10.17632/zrc982rvjm.2 [https://data.mendeley.com/datasets/zrc982rvjms/2]).<br><br>Code availability statement:<br>Source codes necessary for production of the diagnostic- and prognostic-Meningioma epigenetic Liquid Biopsy (dMeLB, pMeLB) classifiers are available at GitHub [https://github.com/gherrgo/eLB-Random-Forests.git]. Complementary *.idat files from tumor tissue and liquid biopsy specimens required to construct, validate, and apply our random forest classifiers are available under the Mendeley Data Accession DOI: 10.17632/zrc982rvjm.2 [https://data.mendeley.com/datasets/zrc982rvjm/2], as well as within the supplementary data (Supplementary Data S1 - Clinical Information; Source Data).

For manuscripts utilizing custom algorithms or software that are central to the research but not yet described in published literature, software must be made available to editors and reviewers. We strongly encourage code deposition in a community repository (e.g. GitHub). See the Nature Portfolio guidelines for submitting code & software for further information.

Data

Policy information about availability of data

All manuscripts must include a data availability statement. This statement should provide the following information, where applicable:

- Accession codes, unique identifiers, or web links for publicly available datasets
- A description of any restrictions on data availability
- For clinical datasets or third party data, please ensure that the statement adheres to our policy

The raw cfDNA methylation intensity data files (EPIC Array; .idat), as well as generated classifiers, have been deposited to Mendeley Data under accession DOI: 10.17632/zrc982rvjm.2 [https://data.mendeley.com/datasets/zrc982rvjm/2]. The Whole genome bisulfite sequencing (WGBS) files generated in this study have been deposited to the Sequence Read Archive at the NCBI under accession code PRJNA932734 [https://www.ncbi.nlm.nih.gov/bioproject/PRJNA932734/]. Additional tumor tissue molecular data analyzed in this study was obtained from Gene Expression Omnibus (GEO) under accession codes GSE42882 [https://www.ncbi.nlm.nih.gov/geo/query/acc.cgi?acc=GSE42882], GSE109381 [https://www.ncbi.nlm.nih.gov/geo/query/acc.cgi?acc=GSE109381], GSE85135 [https://www.ncbi.nlm.nih.gov/geo/query/acc.cgi?acc=GSE85135], GSE189521 [https://www.ncbi.nlm.nih.gov/geo/query/acc.cgi?acc=GSE189521] GSE183656 [https://www.ncbi.nlm.nih.gov/geo/query/acc.cgi?acc=GSE183656], GSE115783 [https://ncbi.nlm.nih.gov/geo/query/acc.cgi?acc=GSE115783], GSE54415 [https://ncbi.nlm.nih.gov/geo/query/acc.cgi?acc=GSE54415], GSE164994 [https://www.ncbi.nlm.nih.gov/geo/query/acc.cgi?acc=GSE164994], GSE147391 [https://www.ncbi.nlm.nih.gov/geo/query/acc.cgi?acc=GSE147391] and GSE111165 [https://www.ncbi.nlm.nih.gov/geo/query/acc.cgi?acc=GSE111165]. Other sources of tissue molecular data employed in this study also include Mendeley Data under accession DOI: 10.17632/5pzd2rg5ys.2 [https://data.mendeley.com/datasets/zrc982rvjm/2] and The Cancer Genome Atlas's GDC data portal [https://portal.gdc.cancer.gov/], as detailed in Table 2. Source of our data is provided with this paper. Public data repositories employed throughout this paper include GENCODE (GRCh38.p12) https://www.gencodegenes.org/human/release_31.html, Infinium Annotation Manifests (hg38) https://zwdzwd.github.io/InfiniumAnnotation, Ensembl (hg38) http://useast.ensembl.org/index.html, and GeneHancer (hg38) https://www.genecards.org/. Gene-ontology and gene-set enrichment was performed using Ingenuity Pathway Analysis (IPA) https://digitalinsights.qiagen.com/products-overview/discovery-insights-portfolio/analysis-and-visualization/qiagen-ipa/.

Human research participants

Policy information about studies involving human research participants and Sex and Gender in Research.

Reporting on sex and gender

Biological sex was annotated at time of sample collection, as reported through either medical records or by the respective published material. In this investigation, biological sex was incorporated and analyzed for association with multiple results, including: unsupervised k-means hierarchical clustering, predicted recurrence risk (p-MeLB), and other supervised analyses. To remove the possibility of epigenetic contributions by differences in X & Y chromosomes, removal of CpG probes associated with either was conducted prior to any and all analyses.
As detailed in Table 1, we had relatively balanced proportions of sex across both meningioma cohorts, with the female sex predominating our tumor tissue cohort (tumor tissue: ~55% [n=49/89] and liquid biopsy (serum & plasma): ~47% [n=39/83]). For the external cohorts (Harmanci et al., 2018, Choudhury et al., 2022, Bayley et al., 2022 and Gao et al., 2013), information regarding sex was available across Bayley's (f: 67, m: 43) and Gao's (f: 14; m: 5) cohorts, only.

Population characteristics

The "original" cohort was comprised of meningiomas (n= 63), gliomas (n= 109), pituitary tumors (n= 14), other CNS tumors (lymphoma, n= 4), and non-neoplastic conditions employed as controls (total=14: abscess, n=2; colloid cyst, n=1; necrosis, n=3; infection, n = 2; epilepsy, n =2; vasculopathy, n=2; non-specific/reactive, n=1; histiocytosis, n=1), further detailed in Table 1. The liquid biopsy serum cohort included a majority of individuals with Caucasian descent, with a median age at diagnosis of 60 and 54.0 years, for meningioma and non-meningioma groups, respectively.
"Additional" liquid biopsy cohorts (MNG: LB-serum: n=20; LB-plasma: n=10; Non-MNG: LB-serum: n=6), used for further validation of machine learning classifiers exhibited similar characteristics including median age (~501 years of age) and majority Caucasian descent.
Included internal tissue cohorts consisted of 66 meningioma collections (both primary [n=40] and recurrent [n=26]), spanning all three WHO grades, primarily taken from patients of Caucasian descent. External tumor tissue cohorts consisted of meningioma collections (Choudhury et al., 2022: 565; Harmanci et al., 2018: 96; Bayley et al., 2022: 110; Gao et al., 2013: 23), spanning multiple WHO grades, further detailed in Table 2.

Recruitment

Patients were recruited from pre-existing patient populations at both the Henry Ford Health (HFH) and University of Sao Paulo (USP) sites. Only patients who provided consent for blood-draw and the use of their samples and associated clinical characteristics for research purposes following tumor diagnosis were included in the study. We only included samples with sufficient material for analysis and profiling. Liquid biopsy samples were collected either during surgery (before tumor removal) or during routine MRI follow-up at the HFH site. We took care to address any potential biases or self-selection issues that could have impacted our retrospective study design.

Ethics oversight

Our research complies with all ethical regulations within our Institution. This project was approved by the Institutional Review Boards (IRB) and patients consented to have their specimens used for research purposes (Henry Ford Health (HFH): IRB#12490; University of Sao Paulo (USP): IRB#1572/2016).

Note that full information on the approval of the study protocol must also be provided in the manuscript.

Field-specific reporting

Life sciences study design

All studies must disclose on these points even when the disclosure is negative.

Sample size	Sample sizes were determined at the start of our investigation from the available retrospective liquid biopsy sample pool, collected between 06/2011 and 08/2019. Sizes were deemed sufficient for formulation of random forest algorithms through application to external cohorts of rivaling or expanded sizes which included samples possessing adequate clinical (person-time) and molecular information. Notably, we incorporated a separately profiled independent cohort, namely "additional" cohort (MNG: liquid biopsy serum [n=20] , plasma [n=10] and tumor tissue [n=39]; Non-MNG: liquid biopsy serum [n=6]) following model derivation, collected between 12/1999 and 07/2021, allowing for further validation of our prognostic findings.
Data exclusions	There were no exclusionary measures taken following formulation of our total internal cohorts. Across correlative analyses (prognostic classification comparisons) using Bayley et al., 2022 at the authors instruction, we excluded samples [n=15] which were not correctly classified by their algorithm. Across similar analyses using Choudhury et al., 2022, we excluded samples [n=60] which were not fully annotated across their provided clinical data.
Replication	Raw cfDNA data for the liquid biopsy collections necessary for reproduction/replication of several analyses have been provided. As mentioned, we profiled additional liquid biopsy serum [n=26], plasma [n=10] and tumor tissue [n=39] following diagnostic/prognostic classifier derivation, in addition to incorporation of external cohorts with prognostically-relevant clinical data (Choudhury et al., 2022 [n=505], Bayley et al., 2022 [n=95]) for replication/validation of our results. Notably, syntax for construction of our formulated diagnostic classifier is available for download, alongside the formulated diagnostic and prognostic classifiers under the accession DOI listed.<br>We conducted several replication attempts of the results for application of the diagnostic and prognostic classifiers across the included raw cfDNA methylation data, with all of them being successful.
Randomization	During formulation of random forest algorithms, machine-driven randomization of relevant cohorts was conducted and weighted by relevant sample types. For example, for diagnostic classification, equivalent distribution of sample types (meningioma, glioma, pituitary tumor, other CNS diseases, and non-neoplastic controls) across discovery (80%) and independent (20%) cohorts was assured for adequate assessment of predictive power. Further machine-driven randomization of the discovery cohort, into training (80%) and model selection (20%) sets, was also incorporated for each iteration. Similarly, for prognostic model derivation, samples were randomized into training and independent sets using a 2/3 split, weighted by absence or presence of a confirmed recurrence to assure equal distributions across model building and validation processes.<br>For experiments other than those mentioned here, there was no randomization of experimental groups, all groups were defined by aforementioned covariates relevant to the experimental design (e.g., diagnostic t-SNE: tumor types as included in Capper et al., 2018; tissue PGP analysis: hypermitotic high risk vs. merlin-intact low-risk MNG). All information relevant to experimental groups has been included in full detail, when relevant, throughout our Methods and Results.
Blinding	Blinding was not relevant to our study design, as this is a retrospective study, not a Randomized Control Trial. We are not administering any sort of treatment, formulating any randomized experimental groups and comparing survival, etc. It was crucial that we remained un-blinded and aware of survival status, recurrence, WHO grade, etc., for each patient, as it informed the interpretation of our results.

Reporting for specific materials, systems and methods

We require information from authors about some types of materials, experimental systems and methods used in many studies. Here, indicate whether each material, system or method listed is relevant to your study. If you are not sure if a list item applies to your research, read the appropriate section before selecting a response.

Materials & experimental systems

n/a	Involved in the study	
☒	☐	Antibodies
☒	☐	Eukaryotic cell lines
☒	☐	Palaeontology and archaeology
☒	☐	Animals and other organisms
☒	☐	Clinical data
☒	☐	Dual use research of concern

Methods

n/a	Involved in the study	
☒	☐	ChIP-seq
☒	☐	Flow cytometry
☒	☐	MRI-based neuroimaging
