# Supplementary material for: Detection of diagnostic and prognostic methylation-based signatures in liquid biopsy specimens from patients with meningiomas
Source: Nat Commun. 2023 Sep 13;14:5669. doi: 10.1038/s41467-023-41434-z (PMC10499807; doi:10.1038/s41467-023-41434-z)
Supplement: Supplementary file 4 — Description of Additional Supplementary Files [file 41467_2023_41434_MOESM4_ESM.pdf]

Description of Additional Supplementary Files

Supplementary Data 1
Description: Clinicopathological and cell type composition estimates through MethylCIBERSORT associated with the serum specimens included in the study.

Supplementary Data 2
Description: Supplementary data related to meningioma diagnostic-related Figure 1 and Supplementary Figure S3.

Supplementary Data 3
Description: Supplementary data related to meningioma prognostic-related Figure 2 and Supplementary Figure S5.

Supplementary Data 4
Description: Supplementary data related to in-silico validation depicted in Figure 4.

Supplementary Data 5
Description: Supplementary information regarding secondary profiling methods (MethylPCR, Whole Genome Bisulfite Sequencing/WGBS).
